# Optimisation of sample storage and DNA extraction for human gut microbiota studies​

**DOI:** 10.1186/s12866-021-02233-y

**Published:** 2021-05-29

**Authors:** Jekaterina Kazantseva, Esther Malv, Aleksei Kaleda, Aili Kallastu, Anne Meikas

**Affiliations:** Center of Food and Fermentation Technologies, Akadeemia tee 15a, 12618 Tallinn, Estonia

**Keywords:** Human gut microbiota, 16S rRNA gene sequencing, Faecal DNA extraction, Faecal sample storage, Illumina iSeq 100, DNA/RNA Shield solution

## Abstract

**Background:**

New developments in next-generation sequencing technologies and massive data received from this approach open wide prospects for personalised medicine and nutrition studies. Metagenomic analysis of the gut microbiota is paramount for the characterization of human health and wellbeing. Despite the intensive research, there is a huge gap and inconsistency between different studies due to the non-standardised and biased pipeline. Methodical and systemic understanding of every stage in the process is necessary to overcome all bottlenecks and grey zones of gut microbiota studies, where all details and interactions between processes are important.

**Results:**

Here we show that an inexpensive, but reliable iSeq 100 platform is an excellent tool to perform the analysis of the human gut microbiota by amplicon sequencing of the 16 S rRNA gene. Two commercial DNA extraction kits and different starting materials performed similarly regarding the taxonomic distribution of identified bacteria. DNA/RNA Shield reagent proved to be a reliable solution for stool samples collection, preservation, and storage, as the storage of faecal material in DNA/RNA Shield for three weeks at different temperatures and thawing cycles had a low impact on the bacterial distribution.

**Conclusions:**

Altogether, a thoroughly elaborated pipeline with close attention to details ensures high reproducibility with significant biological but not technical variations.

**Supplementary Information:**

The online version contains supplementary material available at 10.1186/s12866-021-02233-y.

## Background

Discoveries connecting the human microbiota to a set of human diseases inevitably lead to the development of trustworthy and unbiased technology for characterisation of the structure and dynamics of the human microbiome. An enormous amount of data collected by the European Metagenomics of the Human Intestinal Tract (MetaHIT) and the NIH-funded Human Microbiome Project (HMP) allows to make conclusions about the magnitude and diversity of human gut bacteria, characterise the microbiome of healthy individuals, and perform an important initial step in understanding the contribution of the microbiota to health and diseases.

Despite the increase of scientific data on sequencing bacterial 16 S rRNA-encoding gene, the highly variable microbial composition of the human gut is difficult to interpret and compare between various studies due to the absence of a standard and accepted procedure for the gut metagenomic analysis [1]. The current technology pipeline could be divided into several important stages including sample collection and preservation, a method for microbial genomic DNA extraction, strategy for sequencing library preparation, selection of sequencing platform, bacterial database choice, and bioinformatic pipeline. Undoubtedly, all these stages are paramount for the acquisition of reliable results and their interpretation. However, the data could be biased towards detecting some taxa over the others and thereby lead to wrong evaluation [2].

The latest developments in the next-generation sequencing (NGS) technologies offer higher resolution and accuracy compared to their predecessors and show relative consistency between different platforms. Among the main platforms used for 16 S rRNA gene-based research of microbiota composition, the Illumina MiSeq and Life Technologies Ion PGM are the most commonly used [3]. Launched in the year 2018 by Illumina, iSeq 100 is a simple and affordable benchtop sequencer that enables high-quality results similar to other Illumina platforms and is recommended for 16 S amplicon-based sequencing. Complementary metal-oxide-semiconductor technology and sequencing by synthesis chemistry allow to perform small genome and amplicon sequencing with high accuracy and at a reasonable cost, features that are useful for massive gut microbiota studies.

One of the most important aspects to consider for gut microbiota sequencing is the choice of the variable region of the 16 S rRNA gene [4, 5]. The primers used for amplification may bind to the regions that are not completely conserved among all bacteria and do not have equal affinity for all possible DNA sequences; due to the high similarity between 16 S rRNA gene sequences, they cannot distinguish between closely related species. Moreover, the choice between one or another sequencing region of 16 S rRNA gene is often connected with the availability of the sequencing reagent with the correct read length for a definite platform, as the length of sequenced variable regions differs depending on the primer used. V4 region of 16 S rRNA gene is a good choice for a taxonomic evaluation of microbiota as it yields the most comparable and the least biased results across various pipelines [6–8], and allows to use 300 bp sequences, the crucial feature for the sequencing platform selection. Detailed discussions of all the challenges and limitations in microbiome analysis are well represented in the literature [9–11].

Poor quality or low concentrations of DNA can be successfully amplified by degenerate primers. This enables the sequencing of diverse populations, but the effect of sample preservation media and storage conditions together with the DNA extraction method should be taken into particularly careful consideration. These two parameters are among the most crucial in the case of gut microbiota sequencing as they can introduce systematic errors and therefore were chosen for the thorough evaluation of our workflow.

Currently, various methodologies and commercial kits are in use for gut microbiota sample collection and storage. To prevent the overgrowth of some bacterial species or DNA degradation that causes taxonomical bias, all bacteria in a sample should be inactivated as soon as possible. Immediate freezing, accepted as the best sample collection practice [12–14], is not always possible. Furthermore, the microbiome of frozen faecal samples can change over time[15]. That is why various preservation media that stabilise or fix bacterial gDNA for sample storage are being developed. Among the most widely used fixative solutions for faecal sample preservation are ethanol, PSP kit, SDS, and RNAlater [16]. The results of different studies are often contradictory and inconsistent and depend on the period of preservation [17, 18]. Among others, DNA/RNA Shield™ solution (Zymo Research) has been advised as a powerful DNA stabiliser and microbial inactivator suitable for stool sample preservation and is compatible with the majority of DNA extraction methods with minor inhibitors presence for library preparation workflow [19, 20]. What is important, it allows transporting and keeping samples at ambient temperature for a prolonged time with reproducible NGS results.

Considerable variability in the amount and quality of extracted gDNA from stool samples followed by biased NGS results to a great extent depends on the extraction method used [19, 20]. Moreover, the DNA isolation method must be matched with the sample preservation solution and the original material used. Extensive studies were performed to evaluate different commercial stool DNA extraction kits with a focus on DNA yield, purity, and sequencing output [21, 22]. However, preliminary stool sample preparation together with the collection kit compatibility was out of the research focus. A shift towards one or another bacterial genus could be associated with insufficient bacterial lysis or repeated freezing-thawing steps. Despite the commonly accepted opinion that the interindividual difference possesses a much more significant impact on microbial consortia, variation in the bacterial composition of the same individual introduced by inappropriate storage or DNA extraction methods can drastically affect the whole data analysis [23]. All stages in the gut microbiota analysis need careful consideration and testing to evaluate their impact on the interpretation of the final result.

This study aimed to evaluate the impact of a combination of the most variable and important factors on the final microbiota results and their interpretation, namely, sample collection, preservation and storage, and DNA extraction procedure as a whole pipeline. Also, in our study, we showed for the first time that iSeq 100 platform is a reliable and trustworthy benchtop sequencer suitable for routine microbiota research.

## Results

### Comparison of two commercial kits for faecal metagenomic DNA extraction

**Fig. 1:**
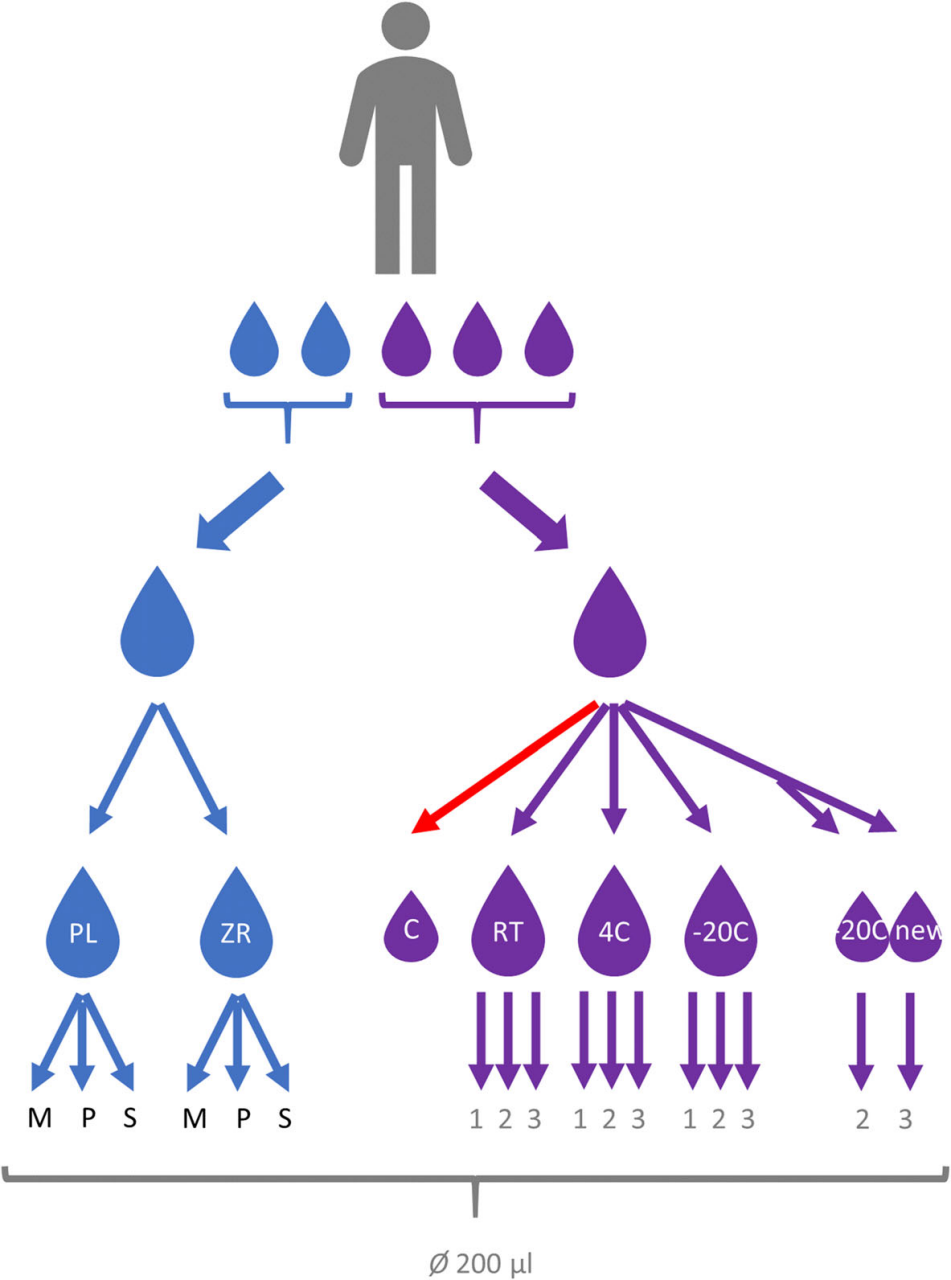
Schematic representation of the experimental design. Blue colour corresponds to the comparison of the commercial kits. PL—PureLink™ Microbiome DNA Purification kit, ZR—ZymoBIOMICS™ DNA Miniprep kit; M—suspension mixture, P—pellet, S—supernatant. Purple colour corresponds to the storage conditions experiment. Marked with the red arrow C—control zero-point sample, analysed within two hours after sample collection. Samples marked with purple arrows were stored at different temperature conditions: RT at room temperature, 4 C at + 4 °C, -20 C at -20 °C (these samples were taken from the same tubes stored at corresponding temperature), -20 C new—two independent aliquots stored at -20 °C and excluded from the freezing-thawing cycle. Samples stored at different temperature conditions were analysed weekly during the three weeks period (1, 2, 3); 200 µl sample aliquots were subjected to the DNA extraction

Five individuals participated in each stage of the experiment (Fig. 1). Their stool was collected at the same conditions and time, vigorously homogenised, and analysed simultaneously. To minimise the variance caused by technical personnel, the same laboratory staff extracted gDNA for all experiments.

For the first validation stage, two available commercial kits were chosen. Both kits, PureLink™ Microbiome DNA Purification Kit (PL) and ZymoBIOMICS™ DNA Miniprep Kit (ZR) were validated for bacterial genomic DNA extraction for microbiome analysis. In addition to the chemical bacterial disruption, microbes were mechanically lysed by bead-beating, as recommended for such studies. Moreover, the PL kit suggests even one more additional heat-lysis step. The application of the multistage lysis approach ensures recovery of microbial DNA from the most complicated samples such as Gram-positive bacteria. Although standard PL protocol suggests gDNA extraction from pelleted stool samples while the ZR kit provides protocol from suspension material, we tested these kits from all possible starting materials, specifically from the pellet (P), suspension mix (M), and supernatant (S). To minimise the possible inhibitory effect of the DNA/RNA Shield™ solution (Zymo Research) for PL extraction protocol, the same amount of cooled sterile phosphate-buffered saline (PBS) was added to the faecal suspension. Samples were prepared similarly for both kits and then treated according to the manufacturer’s recommended procedure. We tested the supernatant remained after centrifugation of the faecal suspension in the DNA/RNA Shield™ solution as it may contain gDNA from residual presence of lysed cells in the PBS-shield solution, which might have lysed during the sample collection, storage, or inside the human gut. We included the supernatant as one of the starting materials for our analysis to better understand the amount and impact of gDNA from lysed bacteria on microbiome test interpretation.

To assess the quality control of the DNA extraction kits and approve the absence of the bacterial contamination from them, additional negative kit-ome controls were done (Suppl. Figure 1), where sterile PBS was used instead of gut microbiome samples. Both kit-ome controls were negative with only 0.0011 or 0.0014 % of the total reads for PL and ZR correspondently. Thereby, all negative controls, including no-template and both kit-omes, performed almost similarly and confirmed cleanliness of the work and low impact of extraction kit impurities on the final sequencing results.

**Fig. 2:**
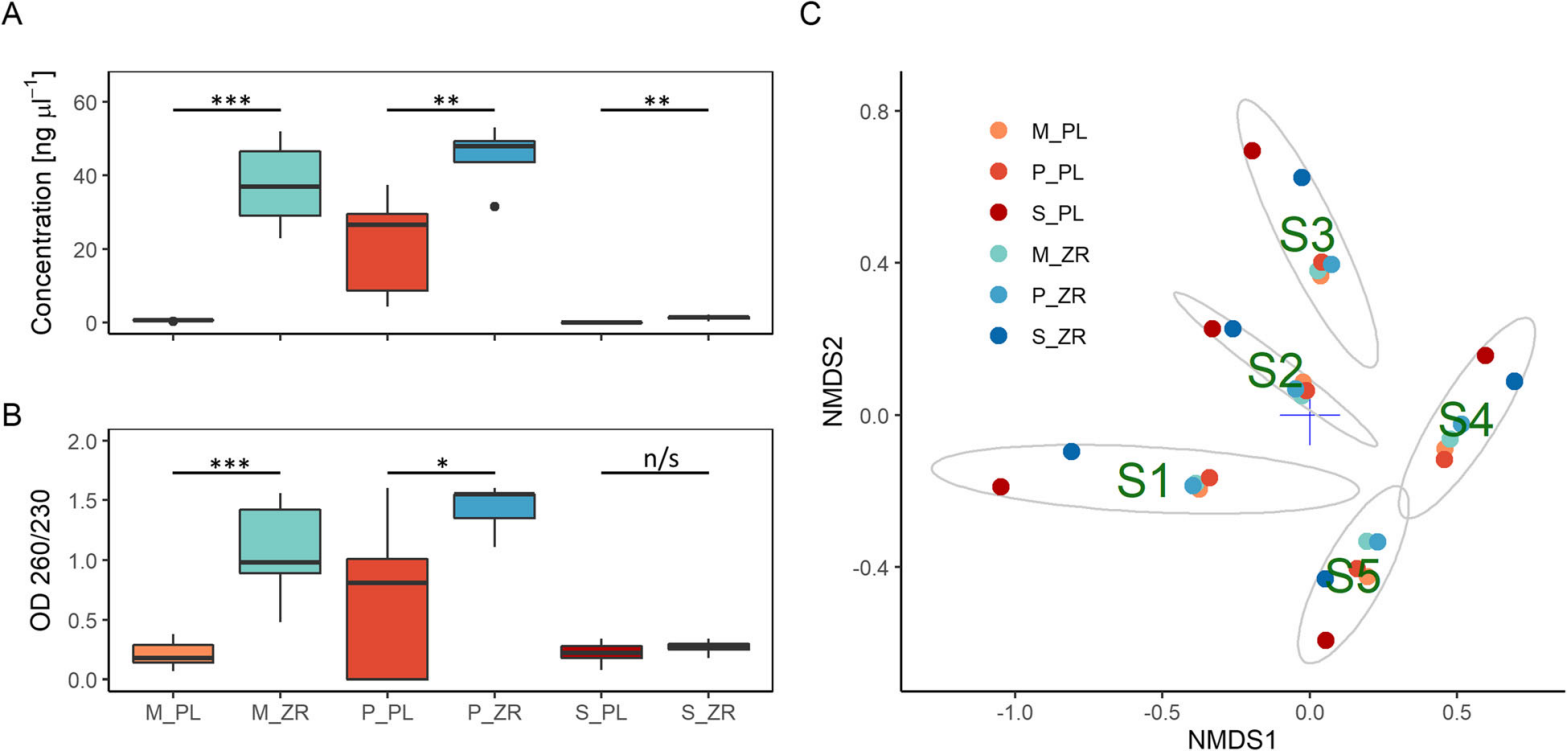
Comparison of different starting material sources (M—suspension, P—pellet, S—supernatant) and two commercial stool DNA extraction kits (PL—PureLink™ Microbiome DNA Purification Kit, ZR—ZymoBIOMICS™ DNA Miniprep Kit) for extracted gDNA (**A**) concentration and (**B**) purity at OD 260/230. Asterisks indicate statistical significance determined by Student’s t-test: *** (*p* < 0.001), ** (*p* < 0.01), * (*p* < 0.05), and n/s not significant. (**C**) Non-metric multidimensional scaling of samples (S1–S5 represent individuals) in response to starting material and isolation kit used. Ellipses indicate distinct clusters of every individual with 95 % confidence

As seen in Fig. 2 A, the total concentration of gDNA obtained by using the ZR kit was higher than for the PL protocol for both pellet and suspension material. Additionally, using only the ZR kit it was possible to extract a substantial gDNA amount from suspension material. Both kits showed a negligible amount of gDNA extracted from supernatants, suggesting an insignificant proportion of consumable contamination for the current collection method used. Furthermore, the quality of extracted DNA checked by the OD 260/230 ratio (Fig. 2 B) was higher in the case of the ZR kit. gDNA integrity checked by the agarose gel electrophoresis (Suppl. Figure 2) indicated high-quality extraction by both kits. Altogether, despite the suitability of both kits for gDNA isolation for microbiota analysis, the ZR kit exhibited better quality characteristics of isolated DNAs.

The next step of microbiota analysis was performed by sequencing the V4 region of bacterial 16 S rRNA on iSeq 100 platform (Illumina). For suspended or pelleted material, the amplicon library was prepared with 12.5 ng of total extracted gDNA that was different for supernatant gDNA, which concentration was much lower. As the amplicon sequencing technology assumes PCR amplification stages that often increase the impact and ratio of low-represented bacterial species, special attention for comparison studies should be put for the equal DNA load during a library preparation, which is not always possible. As a result, the quantitative comparative analysis of supernatant was not equivalent to the pellet and suspension gDNA bacterial composition, representing only a set of lysed bacteria but not their real ratio in the sample.

The non-metric multidimensional scaling (NMDS) of sequenced samples (Fig. 2 C) showed clear grouping by the individual, indicating similarity independent of the isolation kit, or starting material taken for gDNA extraction. However, the bacterial composition of the supernatant samples considerably differed from the pelleted and suspension material. Variation among the samples of one individual was most likely connected to the heterogeneity of the initial material than to the isolation method used.

**Fig. 3:**
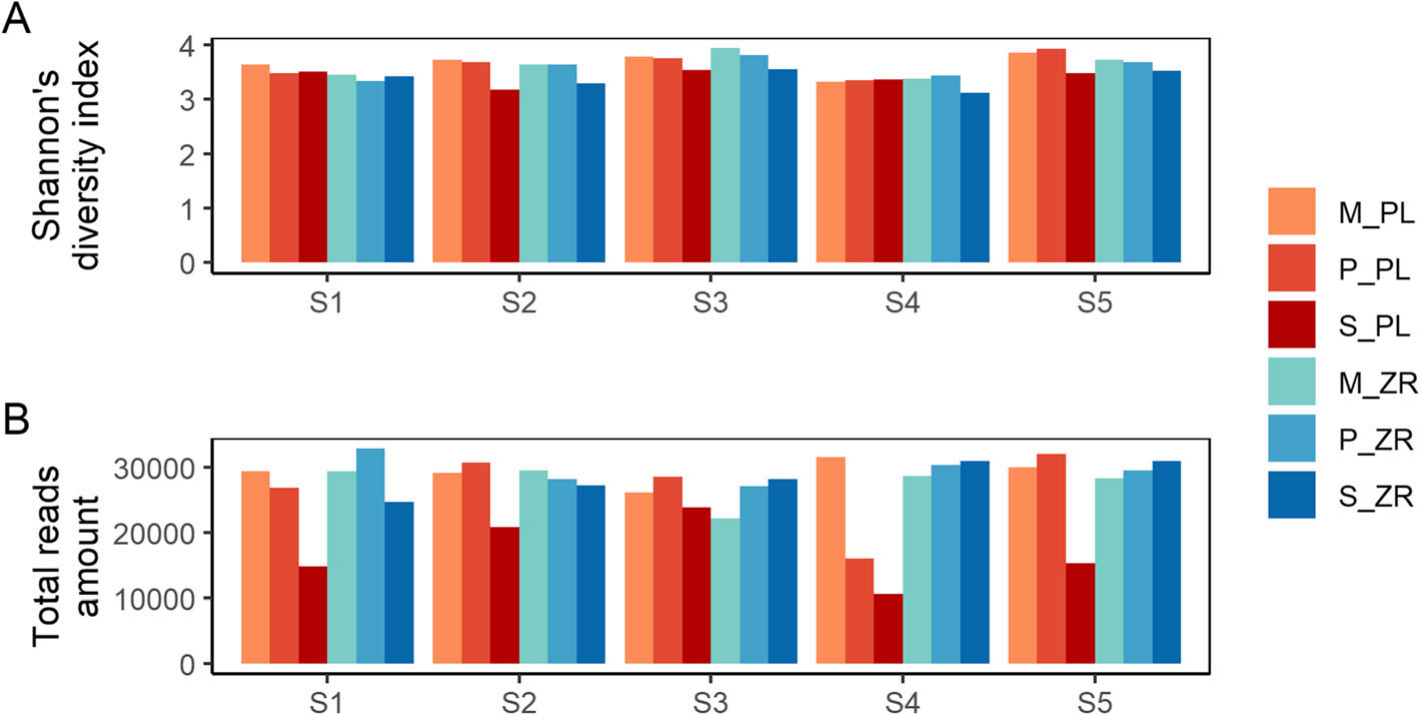
Comparison of (**A**) alpha diversity index (Shannon’s index) and (**B**) the total amount of sequencing reads between samples obtained from different subjects (S1–S5), starting materials (M—suspension, P—pellet, S—supernatant), or DNA isolation kit used (PL—PureLink™ Microbiome DNA Purification Kit, ZR—ZymoBIOMICS™ DNA Miniprep Kit)

Despite the better characteristics of gDNA isolated by the ZR kit, microbiota profiles obtained by both kits were quite similar and it was difficult to prefer one to another extraction protocol. Considering the equal amount of gDNA for library preparation, the alpha-diversity of the gut microbiota assessed by Shannon’s index was similar between suspension and pellet samples for both kits (Fig. 3 A). Supernatant samples were different due to the smaller amount of gDNA taken for the library preparation and consequently showed lower counts of detected bacterial species. Moreover, the number of sequence reads was more reproducible for the ZR isolation method regardless of the starting material (Fig. 3B). Surprisingly, this number was even higher for three out of five supernatant samples. This was different in the case of the PL kit, which had the lowest amount of reads from the supernatant samples. Such a phenomenon can affect the whole interpretation of the results for microbiota studies.

### The effect of material storage conditions on the gut microbiota

ZR DNA extraction kit was chosen for this study stage as it showed slightly more stable and qualitative results and is compatible with the shield collection solution produced by the same company. gDNA was extracted from the suspension mixture of a faecal sample in the shield preservation solution. Faecal samples were stored for up to three weeks at room temperature (RT), + 4 °C, and − 20 °C. The latter was either taken for analysis as a new aliquot (-20 °C new) or repeatedly frozen and thawed (-20 °C).

**Fig. 4:**
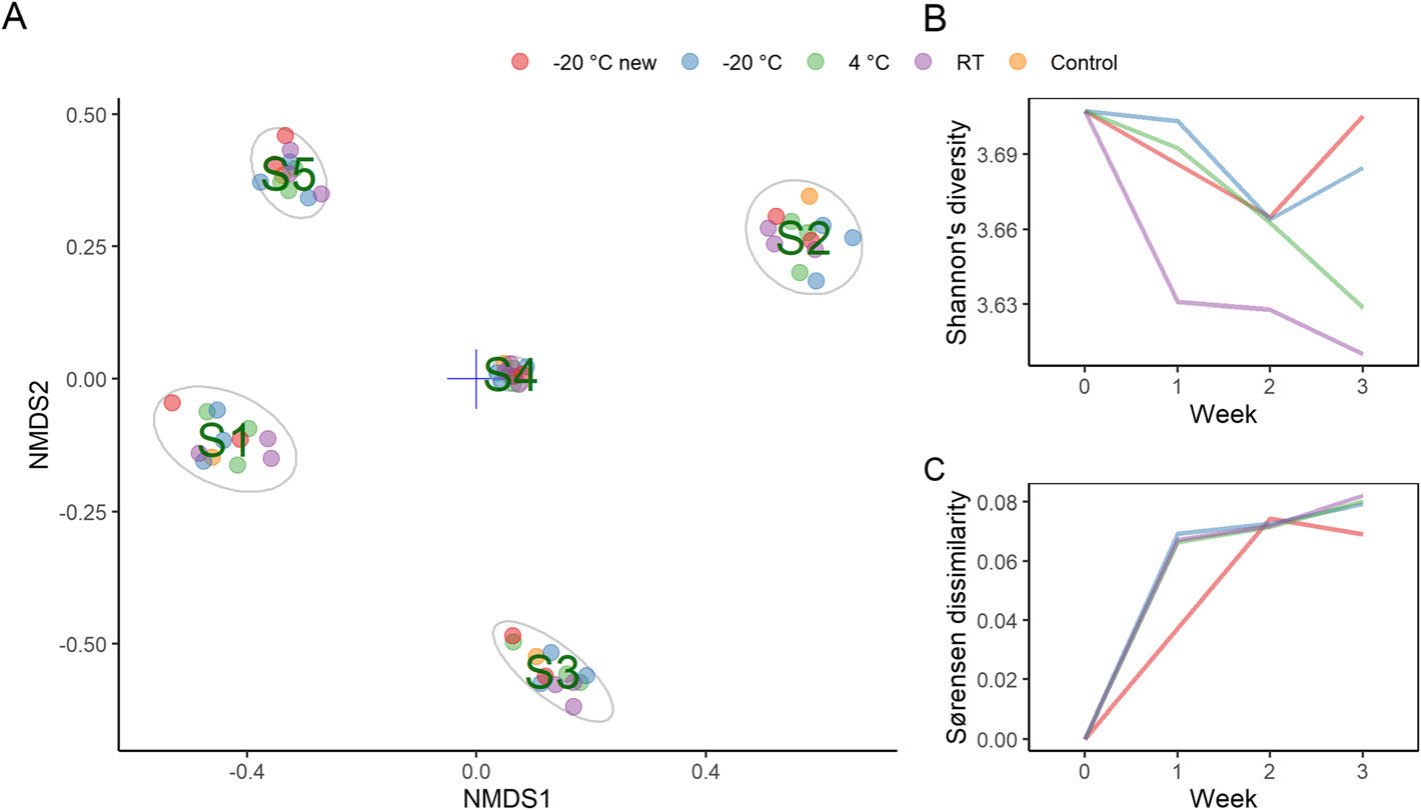
Stability of faecal microbiota under different storage conditions: immediate DNA extraction (Control), at room temperature (RT), + 4 °C, -20 °C new aliquots, repeated freezing and thawing (-20 °C), and up to three-week period represented by (**A**) non-metric multidimensional scaling, where ellipses indicate 95 % confidence, (**B**) Shannon’s alpha diversity, and (**C**) Sørensen dissimilarity index for different tested subjects (S1–S5)

NMDS of sequenced samples kept at various temperature conditions revealed their uniform distribution among the individuals independent of storage parameters, indicating stable results for all tested temperatures (Fig. 4 A). Analysis of the most abundant bacterial strains for each person showed patterns very similar to the controls and uniform across the temperatures and time points. This supports the efficiency of the shield solution to conserve faecal samples at different temperatures during the three weeks of storage. Shannon’s index registered a decline in diversity for samples stored at RT (Fig. 4 B) but considering the magnitude, it was not significant. The storage of samples for one week had almost no impact on the number of detected bacteria for all other temperature conditions. Sørensen dissimilarity index (Fig. 4 C), which was close to zero, also corroborated the stability of the microbial communities during the tested period and confirmed that the repeated freezing and thawing had no significant impact on the microbiota identification. Although Sørensen dissimilarity graph showed high similarity between the control and stored samples, additional calculation of correlations and ANOVA analysis (*р* = 0.5) reinforced high similarity between all the samples.

**Fig. 5:**
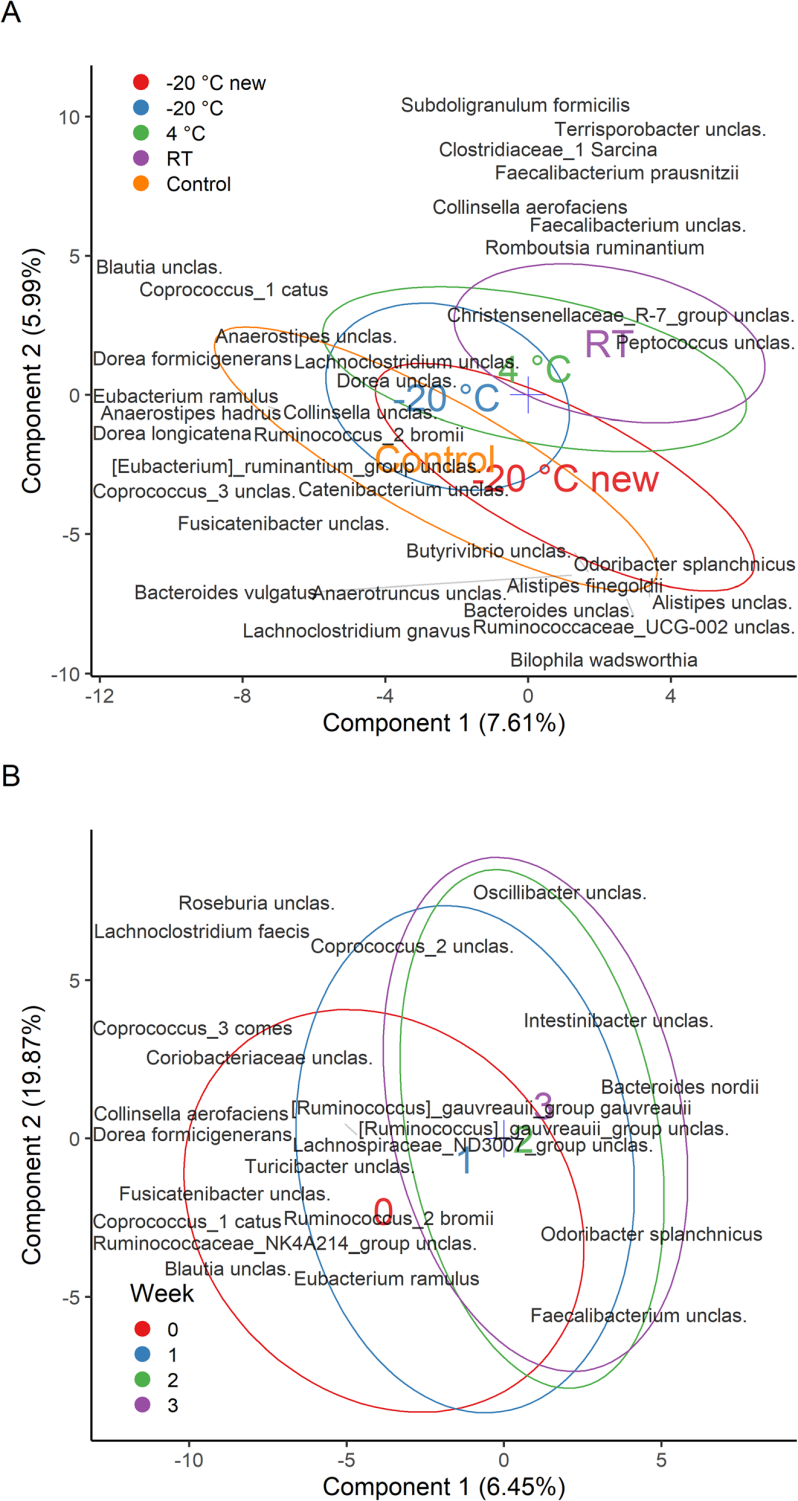
Effect of the (**A**) storage conditions and (**B**) timeline according to the partial least squares (**A**) discriminant analysis and (**B**) regression among the most similar individuals (**A**) S1, S3, and S5, and (**B**) S1–S4. Ellipses indicate 95 % confidence range. Only the most important species for the model are shown. The numbers in parentheses show the variance explained by the component

Partial least squares discriminant analysis and regression were applied to investigate the differences between the samples in more detail. These algorithms identify features that vary between the treatments, in this case, the storage conditions and the time (Fig. 5). Overall, there were no common differences between the tested storage conditions, nevertheless, a model built using the data of three out of five most similar individuals (S1, S3, S5) presented some trends (Fig. 5 A). Only the most important species are shown (variable importance in projection > 1.2) on the plot. Considering the divergence between these biological samples, the results revealed that storage at -20 °C had the smallest impact on the microbiota composition compared to the control. The higher the temperature of the storage, the further away the cluster was from the control, indicating changes in the microbial profile. Nevertheless, these changes were minor in absolute terms, as confirmed also by the low explained variance of the two components (13.6 %).

Similarly, the partial least squares regression analysis indicated that the temporal dimension (up to three weeks) had low effect on the sample storage in aforementioned conditions using data of individuals S1–S4 (Fig. 5 B). Abundance of some bacteria like *Oscillibacter* spp., *Intestinibacter* spp., and *Bacteroides nordii* increased during storage, while *Blautia* spp. and *Eubacterium ramulus* decreased, but such trends were few and of little importance, as confirmed by the low variance explained by the first component (6.45 %).

**Fig. 6:**
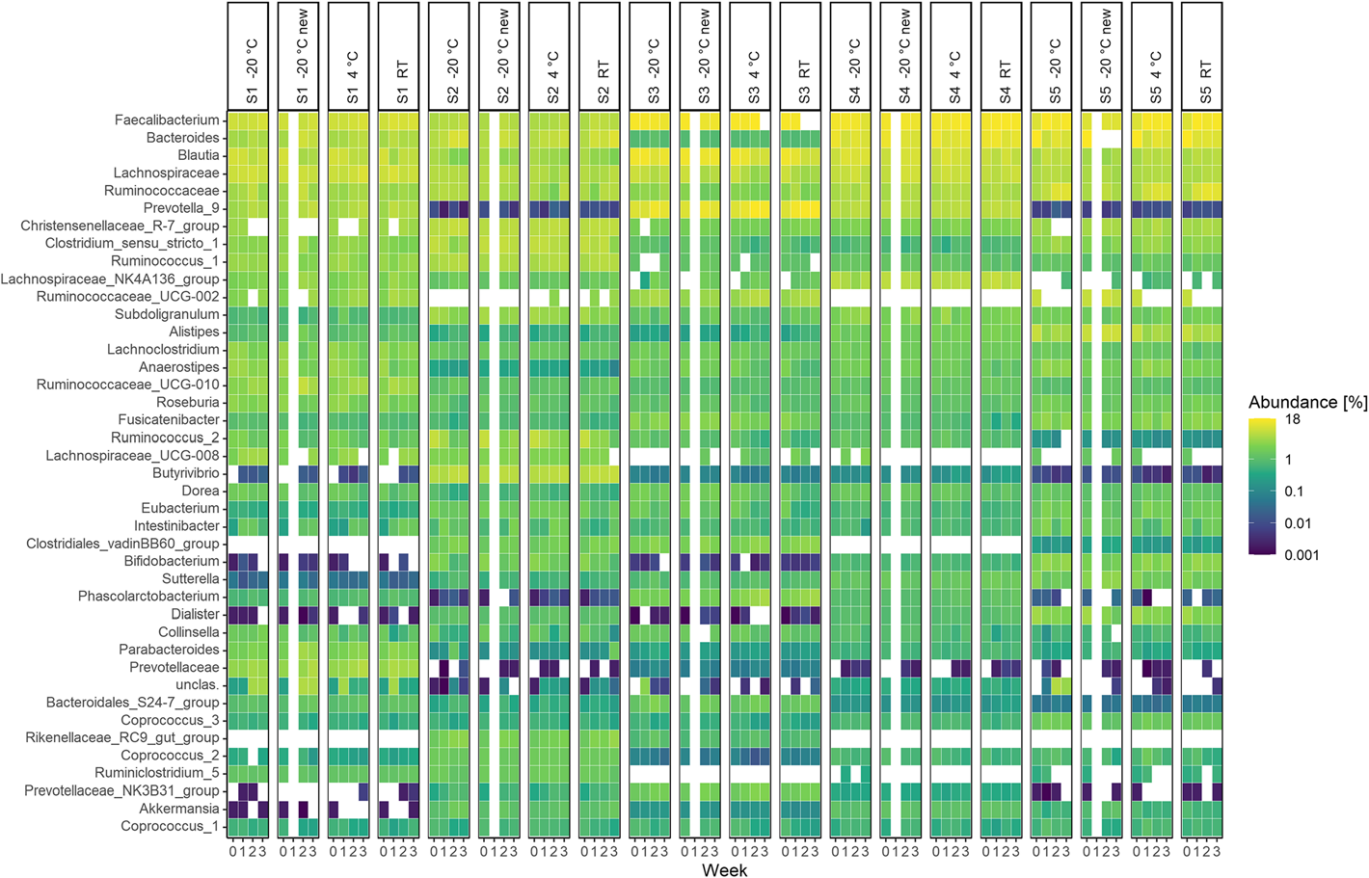
Heatmap of the abundance of faecal bacteria under the different storage conditions: at room temperature (RT), + 4 °C, -20 °C new aliquots, repeated freezing and thawing (-20 °C), and up to three-week period for the different tested subjects (S1–S5). Numbers (0–3) below the heatmap correspond to the week of storage, where 0 is the control. The yellow colour in the heatmap indicates a high number of bacteria in the sample, while the dark blue corresponds to their low abundance. Blank indicates the absence of data at this point. Only 40 of the most represented bacterial genera or familiae across all individuals are shown

Next, a heatmap analysis was performed to visualise the sequencing data (Fig. 6). As the gut microbiotas we identified had more than 440 different bacterial species (Supplementary Table 1), we analysed the samples on their genus or, in the case of unidentified genus, on the family levels. Only 40 of the most abundant genera or familiae were included into the heatmap. The most represented bacterial genus in all individuals belong to *Faecalibacterium* followed by *Bacteroides* and *Blautia*. Again, the abundance of identified bacteria was stable among biological individuals independent of the storage time and conditions. As seen in Fig. 6, some individuals, such as S4, had a more constant distribution of bacteria compared to the control, whereas others (S2 and S5) were more divergent at the different storage conditions. However, in general, we can claim that keeping faecal samples at + 4 °C or -20 °C for one week in the shield solution had minimal impact on the microbiota composition. Additionally, repeated freezing and thawing procedure did not change dramatically the whole bacterial distribution, indicating that the same sample can be repeatedly used in case a re-test is needed.

**Fig. 7:**
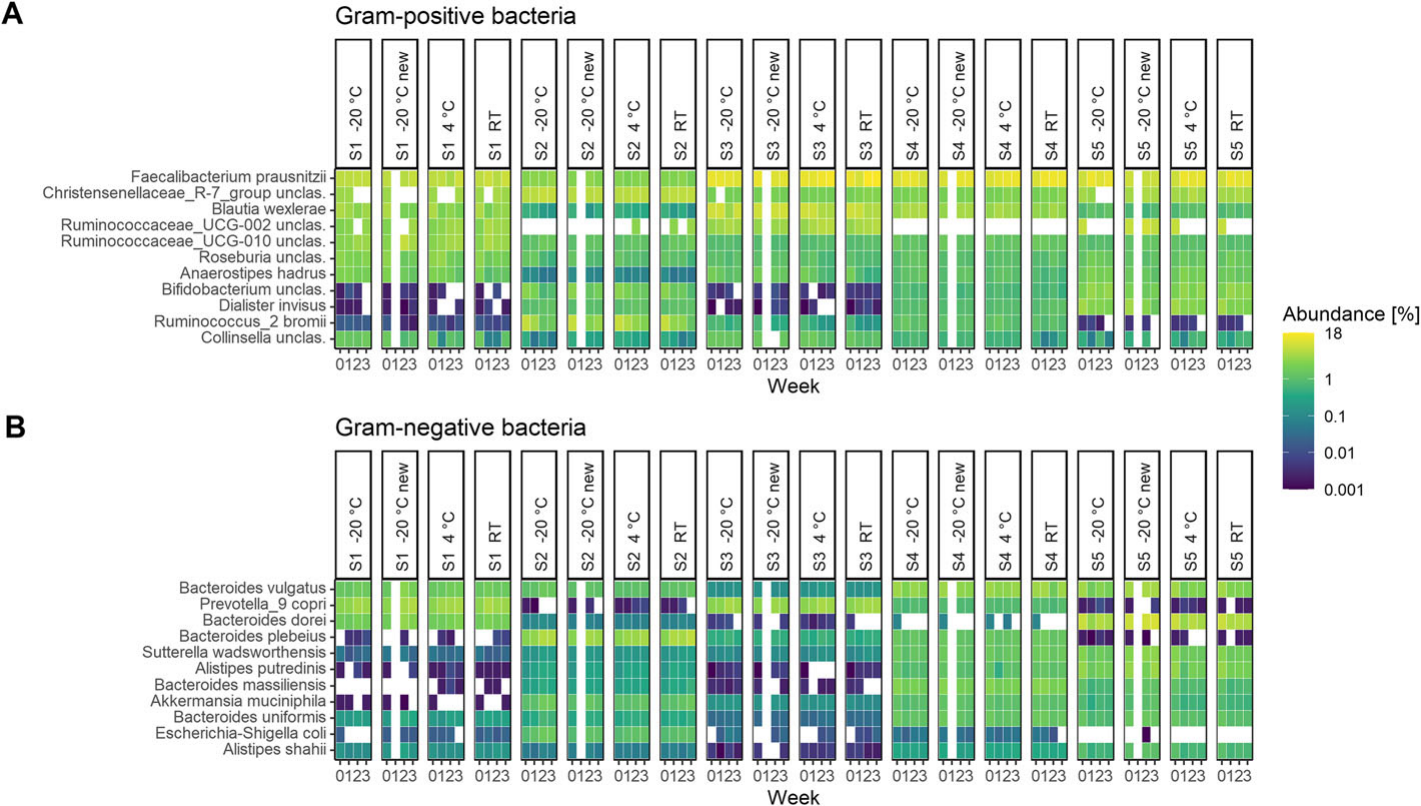
Heatmap of abundances of some selected (**A**) Gram-positive and (**B**) Gram-negative faecal bacteria under the different storage conditions: at room temperature (RT), + 4 °C, -20 °C new aliquots, repeated freezing and thawing (-20 °C), and up to three-week period for the different tested subjects (S1–S5). Numbers (0–3) below the heatmap correspond to the week of storage, where 0 is the control. The yellow colour in the heatmap indicates a high number of bacteria in the sample, while the dark blue corresponds to their low abundance. Blank place indicates the absence of data at this point

Gram-positive bacteria have a thick peptidoglycan layer in their cell wall that makes them more difficult to lyse than most Gram-negative bacteria, which can influence the whole microbiome analysis. To understand the specific effect of storage conditions on Gram-positive and Gram-negative bacteria, several most abundant bacterial species from these categories were selected and visualised as heatmaps (Fig. 7 A, B). This analysis also did not show any evident negative impacts of sample storage conditions or time. A slight reduction of Gram-negative and an increase of Gram-positive bacteria during the storage period were observed for individuals S4 and S5. At the same time, S2 and S3 showed the opposite tendency, whereas the distribution between Gram-positive and Gram-negative bacteria in S1 fluctuated in response to the storage conditions. Altogether, these data indicate low impact of temperature and temporal parameters on the detection shift between various bacteria groups.

## Discussion

Proper handling of stool samples and reproducible and reliable gDNA extraction is crucial for the gut microbiota studies. In this work, we investigated the impact of various storage conditions, variations in starting material sources, and two commercial DNA extraction kits on the composition of bacterial communities in faecal samples collected into the DNA/RNA shield solution. We approved the usage of Illumina iSeq 100 System as a reliable platform for the performance of human gut microbiota studies by sequencing the V4 region of the 16 S rRNA gene.

Despite their high bacterial load, stool samples are considered a challenging material due to their high heterogeneity, variable composition, and large amounts of well-known PCR inhibitory substances such as bilirubin, complex polysaccharides, and some types of lipids [24]. The addition of sample washing or dilution steps by commonly used buffers such as PBS and pre-clearing of the initial material by centrifugation address the issue and is a common way to improve the quality parameters of gDNA extraction. When investigating the bacterial composition of the human gut, delay between sample collection and DNA extraction can allow selective growth of some species at the expense of others [25]. Thus, the method of sample collection and preservation together with the storage temperature and time are crucial for the adequate interpretation of the results. A uniform pipeline with compatible components that minimises biases and unpredictable outcomes is paramount for the whole analysis interpretation.

For our microbiota study, we collected human stool material using the DNA/RNA Shield™ collection tubes by Zymo Research to inactivate and stabilise microbes from human faeces. The manufacturer Zymo Research claims that the patented composition of the shield solution conserves the DNA content of the collected samples at ambient temperature for more than one year, while samples in the shield solution can be frozen (-20/-80 °C) for longer periods without any observed significant changes in microbial composition. In our study, we decided to evaluate the efficacy of the DNA/RNA Shield™ solution for different options of faecal material sample storage. Other researchers estimate that despite the satisfactory results of commonly used and validated commercial preservation solutions, their effect on the stability of the gut bacterial community is temperature- and time-dependent and should not be ignored. Thus, several studies of collecting and storing gut microbiome samples in RNAlater [26], *OMNIgene GUT* [13], below − 20 °C [15, 27], or at ambient temperature using “laboratory kits” [28] investigated the effect on microbiota composition. In general, the technical variability between various conservation methods is crucial but remains smaller than the inter-individual variability, which is also supported by our results. Our study confirmed that the commercially available shield solution is suitable for gut microbiota analysis and adequately performs its functions. Even at the species level, fluctuations in bacterial composition during the various storage conditions, times and repeated freeze-thawing cycles were not significant, which makes the DNA/RNA Shield an excellent choice for sample conservation for microbiome testing. Furthermore, its composition inactivates pathogenic microbes and viruses that could be presented in stool samples, bringing additional value for biosafety during sample transportation and handling by the laboratory personnel, especially in the light of the COVID-19 pandemic, as it has been shown that this virus can persist in stool [29].

While the impact of each step in the sequencing-based analysis of the microbiome is likely important, it has been widely accepted that the DNA extraction method plays a central role in the fluctuation and representativity of complex bacterial communities [30, 31]. As the gut microbiota consists of both Gram-positive and Gram-negative bacteria and their relative abundances span several orders of magnitude, it is important to choose an unbiased gDNA extraction method that can resolve these tasks and systematically implement reproducibility within and across various laboratories. It was shown that mechanical lysis by bead-beating was positively associated with bacterial diversity and is a necessary stage to efficiently extract the DNA of Gram-positive bacteria [21, 22].

Both commercially available kits we tested use bead-beating technology together with chemical lysis and provide reliable and comparable results for gut microbiota analysis. To adequately compare these two DNA extraction kits, we examined different starting material sources of stool samples collected into the shield solution. To minimise the inhibitory effect of the shield preservation solution, we added an equal amount of cold sterile PBS to the samples before the extraction step. As the PureLink protocol allows custom modifications of sample preparation, we decided to choose the pelleted stool as a starting material that is free from possible inhibitors for gDNA isolation and included it for both extraction procedures. Furthermore, the supernatant from the pelleted material can contain the DNA of lysed bacteria and be an informative resource for the shield efficacy. Our results showed that both stool pellet from faecal suspension in the shield together with the suspension itself are equally suitable as a starting material for the human gut microbiota test, representing highly similar compositions of bacterial consortia. Moreover, both kits were comparable in the number of isolated bacterial species. Most likely the difference between these two kits is more significant only in the case of low microbial load samples. A more comprehensive analysis of individual bacterial species might uncover further discrepancies between the kits used, but to make a reliable conclusion, it is necessary to analyse a substantial number of participants and perform systematic statistical analysis. Besides, our personal experience with the PL kit usage in different laboratories revealed its low lab-to-lab reproducibility of gDNA extraction from the same sample. Overall, despite the higher gDNA amount and the better quality parameters for the ZR kit, the biological variability between individuals was much more significant than the observed differences in gDNA isolation technology. In general, both methods did not result in considerable differences according to Shannon’s diversity index while the ZR isolation kit provided more reproducible read counts across the various starting materials. The only observed preference between the two kits in favour of ZR was noticed by the inclusion of the supernatant samples, which were not incorporated in further microbiota profile analysis. Although the amount of DNA from residual lysed bacteria in the supernatant was insignificant, the extraction of total gDNA from suspension material has its advantages. Suspension material gives a comprehensive overview of the whole microbial community of the gut, which in some cases, such as long storage at a higher temperature, could be more representative than the pelleted sample. Since Zymo Research protocol provided the better quality and higher concentration of isolated gDNA, this kit and the faecal suspension were chosen for the next optimisation study.

We have not observed any significant influence of the tested sample storage conditions and time on the final gut microbiota composition. Worth noticing, the minimal differences from the controls showed the samples stored at -20 °C. To our surprise, even repeated freezing and thawing had no meaningful impact on the balance of Gram-positive and Gram-negative bacteria distribution. Thereby, once more our study stresses the greater variability between biological individuals and the possible influence of each matrix on the final results. It is possible to speculate, however, whether the degree of bacteria conservation regulated by faeces/shield ratio is important, or the biochemical composition of the matrix has the strongest impact on the bacterial distribution in response to the storage. Furthermore, however insignificant, the biggest difference in the microbiota composition inside the sample occurred during the first week of storage. Thus, relevant conclusions and reproducible data can be obtained from the samples stored up to three-weeks.

It is worth noting that our results concerning the impacts of bias-generating steps, such as DNA extraction methods and storage conditions, should be interpreted within the context of used kits and conditions only and taken into consideration during the planning of the whole analysis pipeline.

## Conclusions

In the current study, we showed that launched in the year 2018 iSeq100 together with the open-source BION-meta package are suitable and reliable platforms for 16 S rRNA gene-based microbiota analysis. We approved that the DNA/RNA shield is compatible with DNA extraction kits from different manufacturers and is good for different temperature and temporal conditions, providing reproducible results during repeated freezing-thawing procedures. Our experiment showed that the shield solution conserves faecal microbes without lysing the cell membrane, while various stool starting materials, such as a faecal suspension in the shield solution and its pellet, could be taken for DNA extraction. Overall, two tested commercial kits, stool storage conditions and time up to three weeks had only limited effects on the microbiota composition of human faeces samples, stressing the importance of biological but not technical variation. Elaborated amplicon-based 16 S rRNA gene metasequencing pipeline with meticulous attention to small details is crucial for researchers working in the field and extends understanding of all stages of the microbiota research. The standardisation of the microbial meta-sequencing data and the absolute quantification of the bacteria are next most important challenges to resolve in the field of the gut microbiome research now.

## Methods

The full scheme of the study design is shown in Fig. 1.

### Collection of faecal samples

Five faecal samples were collected from healthy adults (mean age 48 ± 8). Written informed consent to participate in the research study was taken from each participant. The faecal samples were gathered with the DNA/RNA Shield Collection Tubes with Swabs (Zymo Research, Irvine, CA, USA) using FecesCatcher by TagHemi (Zeijen, The Netherlands) according to the manufacturer’s instructions. Each collection tube was pre-filled with 1 mL of DNA/RNA Shield™ solution. Sampling material from three different parts of the stool was collected to provide a representative microbial profile. Instruction about aseptic sampling was provided and followed by the participants. Five tubes with pooled faecal material from each participant were used for the experiments. To minimise the effect of samples heterogeneity, the material from different tubes of one individual was put together, thoroughly mixed, and used for the following analyses according to the protocol. All methods were carried out in accordance with relevant guidelines and regulations. This study complied with the Declaration of Helsinki and was approved by the Center of Food and Fermentation Technologies Review Board.

### Sample Storage

For comparison of the two different DNA extraction kits, two faecal samples from each participant were collected using DNA/RNA Shield collection kit and kept at + 4 °C until the analysis. Before DNA extraction, the samples were held at -20 °C overnight. Thereafter, the samples were subjected to DNA extraction according to the protocols.

For the evaluation of the influence of the sample storage conditions, three faecal samples from each person were collected using 1 ml tube of DNA/RNA Shield collection kit and pooled together. Then the pooled sample was divided into aliquots and processed according to the scheme (Fig. 1). The first aliquot was immediately subjected to DNA extraction (Control). The second aliquot was left to the room temperature (RT), the third was stored at + 4 °C. To study the effect of repeated freezing-thawing on the microbiome profile, the fourth aliquot was kept at -20 °C for up to three weeks, from which 200 µl of the material was taken every week for DNA extraction. The fifth aliquot of the sample was divided into two different tubes and kept at -20 °C. Starting from the second week, freshly thawed aliquot was used for the analysis (-20 °C new).

### Microbial Metagenomic DNA Extraction

Two commercial DNA extraction kits were evaluated: PureLink™ Microbiome DNA Purification Kit (PL, Invitrogen, Waltham, MA, USA) and ZymoBIOMICS™ DNA Miniprep Kit (ZR, Zymo Research, Irvine, CA, USA). Samples were prepared similarly for both kits. Before the initiation of DNA extraction, stool samples were thawed at room temperature for 15 min. Two collected samples from one participant were pooled and aliquoted thereafter into two separate tubes. Each aliquot was mixed with an equal amount of cooled sterile 1xPBS (BIO-RAD, CA, USA) and proceeded according to the experiment scheme. PBS was used to minimise a possible inhibitory effect of the shield solution (Zymo Research) for PL extraction protocol. 200 µl of the faecal solution was taken for each separation method. For negative kit-ome analysis, 200 µl of sterile PBS only was taken and carried through the whole cycle of DNA extraction. The first aliquot of faecal material in the shield solution diluted by PBS was considered as a suspension mix (M). For the second aliquot, additional centrifugation at 14 000 × g for 10 min was implemented. The supernatant (S) and pellet (P) were used separately for the following DNA extraction. According to the applied PL or ZR method, 800 µl of corresponding lysis buffer or lysis solution was added to the M, S, or P samples. Subsequent DNA extraction steps followed the PL or ZR kit protocol.

For evaluation of storage conditions, ZymoBIOMICS™ DNA Miniprep Kit was used. Samples were stored as mentioned before and 200 µl of the faecal solution from one subject were used for DNA extraction.

The quantity of the extracted gDNA was measured by Qubit™ 4 Fluorometer (Thermo Fisher Scientific, Waltham, MA, USA) using dsDNA BR Assay Kit (Thermo Fisher Scientific). The quality of the gDNA was assessed by BioSpec-nano (Shimadzu Corporation, Tokyo, Japan) determining the ratio of absorbance of 260 nm to 280 nm, and 260 nm to 230 nm.

### 16S Library preparation and sequencing

The PCR for amplification of the V4 hypervariable region of the 16S rRNA gene of the extracted microbial gDNA was performed using forward primer F515 5’-GTGCCAGCMGCCGCGGTAA-3’ and reverse primer R806 5’-GGACTACHVGGGTWTCTAAT-3’ [8]. The reaction was carried out in 25-µl volume containing 12.5 ng of gDNA, 5 pmol of both primers (Biomers, Germany), and FIREPol® Master Mix with 7.5 mM MgCl_2_ (Solis BioDyne, Estonia). The amplification was performed in an Eppendorf Mastercycler (Eppendorf, Hamburg, Germany) applying the following parameters: preliminary denaturation at 95 °C for 12 min; 20 cycles of denaturation at 95 °C for 30 s, annealing at 55 °C for 30 s and elongation at 72 °C for 30 s; final elongation at 72 °C for 5 min. For the multiplexing, Nextera XT Index Kit v2 Set A (Illumina, San Diego, CA, USA) was used. PCR products were purified by NucleoMag® NGS Clean-up and Size Select Magnetic Beads (MACHEREY-NAGEL, Dueren, Germany) in accordance with the manufacturer’s manual. The quantity of the purified library was determined by Qubit™ 4 Fluorometer using dsDNA HS Assay Kit (Thermo Fisher Scientific). The libraries were pooled at equal molar concentration and sequenced on iSeq100 Sequencing System (Illumina, San Diego, CA, USA) using iSeq 100 i1 Reagent and 2 × 150 cycles paired-end sequencing protocol. Microbial community standard, library stability and reproducibility controls were included into the sequencing run for checking quality and persistency parameters.

### Sequencing data processing

DNA sequence data were analysed as published before [32–34]by BION-meta program (https://github.com/nielsl/mcdonald-et-al) according to the author´s instructions. Sequences were cleaned at both ends using a 99.5 % minimum quality threshold for at least 18 of 20 bases for 5′-end and 28 of 30 bases for 3′-end, joined, followed by removal of shorter contigs than 150 bp. Then, sequences were cleaned from chimaeras and clustered by 95 % oligonucleotide similarity (k-mer length of 8 bp, step size 2 bp). Finally, consensus reads were aligned to the SILVA reference 16 S rRNA database (v123) using word length of 8 and similarity cut-off of 90 %. The bacterial designation was analysed at different taxonomic levels down to species if applicable (Suppl. Table 1).

### Data analysis

Statistical analysis and visualization were done in R version 4.0.2 (R Foundation for Statistical Computing, Vienna, Austria). Package ‘vegan’ 2.5-6 was used for non-metric multidimensional scaling, calculating Shannon’s diversity index and Sørensen dissimilarity. Partial least squares discriminant analysis was performed with package ‘mixOmics’ 6.11.33, and partial least squares regression with package ‘pls’ 2.7-3, data were centred and scaled. Figures show only bacteria with variable importance in projection above 1.35.

## Supplementary Information

**Additional file 1.**

**Additional file 2.**

## Data Availability

The datasets generated and/or analysed during the current study are available in the SRA-NCBI repository, the reference is SRA/PRJNA668920. The datasets supporting the conclusions of this article are included within the article and its additional files. All additional data could be provided upon request.

## Abbreviations

COVID-19: Corona Virus Disease
gDNA: Metagenomic DNA
HMP: Human Microbiome Project
MetaHIT: Metagenomics of the Human Intestinal Tract
NGS: Next-generation sequencing
NMDS: Non-metric multidimensional scaling
PSP: Pre-analytical Sample Processing Spin Stool DNA Kit
SDS: Sodium dodecyl sulphate
