# Supplementary material for: Optimisation of sample storage and DNA extraction for human gut microbiota studies​
Source: BMC Microbiol. 2021 May 29;21:158. doi: 10.1186/s12866-021-02233-y (PMC8164492; doi:10.1186/s12866-021-02233-y)
Supplement: Supplementary file 1 — Additional file 1. [file 12866_2021_2233_MOESM1_ESM.docx]

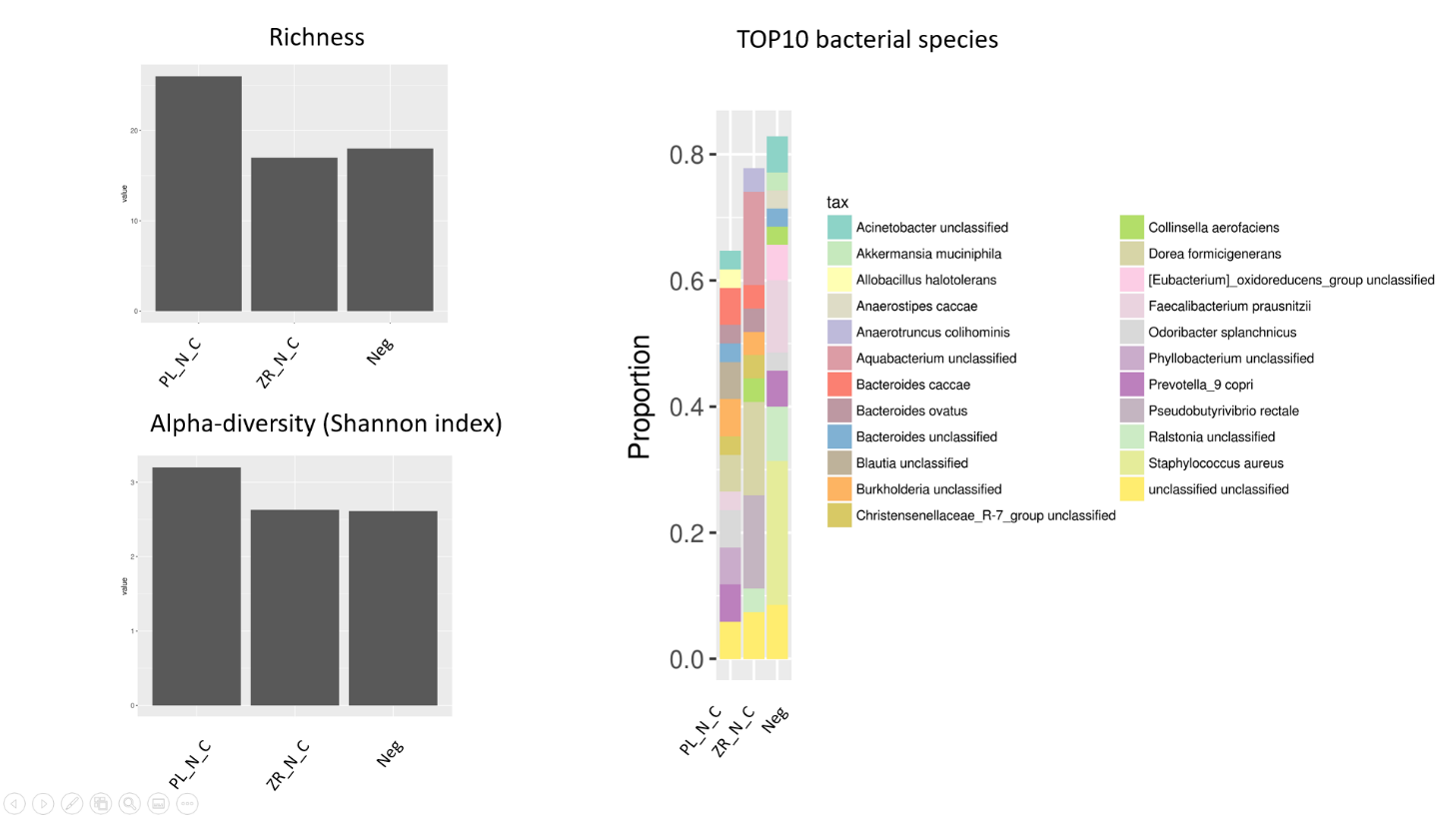


**Supplementary figure 1.** Comparative analysis of all negative controls (kit-ome and no-template sequence control) used in the current study. Data demonstrated as Richness and Alpha-diversity histograms, TOP10 of the most representative bacterial species are shown. No template library negative control (Neg) has got 43 reads (0,0013% from all reads), PureLink negative kit-ome sample (PL_N_C) has got 39 reads (0,0011% of all reads), and ZymoResearch negative kit-ome sample (ZR_N_C) has got 34 reads (0,0014% of all reads), while the average number of reads per sample in the sequencing run was 193200.

**Supplementary figure 2.** Image of the agarose gel for gDNA integrity visualisation. PL - PureLink™ Microbiome DNA Purification kit, ZR – ZymoBIOMICS™ DNA Miniprep kit; 1-5 – different samples. 50 ng of gDNA was put into a well. Thermo Scientific GeneRuler 1 kb DNA Ladder was used as a length marker. Agarose gel electrophoresis conditions were as follows: 0,75 % agarose gel, electrophoresis voltage 70 V and duration 45 min.


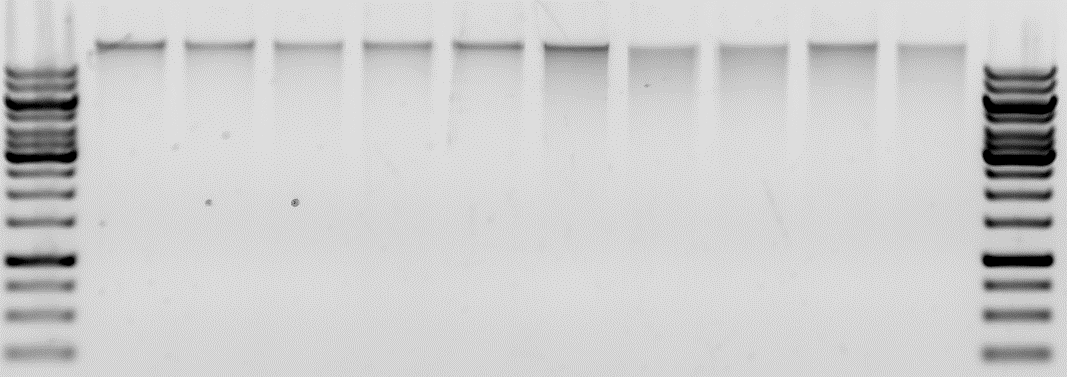


1 2 3 4 5 1 2 3 4 5

PL ZR

bp

10000

6000

3000

1000
